# Gendered Racial Microaggressions and Depressive Symptoms Among Emerging Adult Asian American Women: Racial/Ethnic Differences Among Asian and White Romantic Partners

**DOI:** 10.1007/s40615-024-02184-w

**Published:** 2024-09-30

**Authors:** Michele J. Wong, Brian TaeHyuk Keum, Mary Nguyen, Jung Yun Na

**Affiliations:** 1 Divsion of Social Sciences, University of California, 405 Hilgard Avenue, Los Angeles, CA 90095 USA; 2 Department of Counseling, Developmental & Educational Psychology, Boston College, Boston, MA USA; 3 Department of Social Welfare, University of California, Los Angeles, CA USA; 4 Department of Psychology, University of Maryland, Baltimore County, Baltimore, MD USA

**Keywords:** Asian American women, Gendered racial microaggressions, Asian fetishism, Interracial partnerships, Depressive symptoms, Partner race/ethnicity

## Abstract

Discrimination can contribute to adverse mental health outcomes among individuals in romantic partnerships. However, research has yet to examine how differences in partner race/ethnicity can shape the link between gendered racial microaggressions, an intersectional form of discrimination, and depressive symptoms among Asian American women. Accordingly, we assessed the link between gendered racial microaggressions and depressive symptoms, and whether partner race/ethnicity (White vs. Asian) moderated the link. Using a sample of 156 Asian American women (*M*_age_ = 26.5, *SD* = 5.33), we conducted multiple regressions to assess the main effects between four gendered racial microaggression stress subscale factors and depressive symptoms. We then examined partner race/ethnicity as a moderator in these associations. All four gendered racial microaggression stress subscale factors of ascribed submissiveness, assumptions of universal appearance, Asian fetishism, and media invalidation significantly predicted greater depressive symptoms. However, only Asian fetishism experiences maintained a significant and positive association with depressive symptoms for Asian American women with White male partners. The association between Asian fetishism and depressive symptoms was no longer significant for Asian American women with Asian male partners. Results indicate that Asian fetishization may be a uniquely oppressive experience for Asian American women with White partners that can contribute to greater depressive symptoms. These findings demonstrate an increased need for the development of critical consciousness in individual and couples counseling sessions to help Asian American women and their romantic partners identify and mitigate the negative effects of gendered racial microaggressions.

Romantic partnerships may be especially beneficial for the mental and physical well-being of racially/ethnically minoritized individuals experiencing racism-related stress [1–3]. As prior research suggests, racism-specific support can provide a safe setting for racially/ethnically minoritized couples to share, reassess, and validate the pain associated with experiencing racial discrimination, thereby improving their overall health [1, 2]. Accordingly, the race/ethnicity of romantic partners may be an important indicator for a variety of factors (e.g., shared experiences and understanding of racial discrimination) that can influence the quality of support experienced in the context of racism-related stress, and any potential health benefits. However, few studies have investigated how the racial/ethnic status of romantic partners shape Asian American women’s (AAW) perceptions of gendered racial microaggressions (GRM), a unique intersectional stressor experienced as subtle everyday expressions of denigration based on the simultaneous and compounding experience of racism and sexism [4–6].

Although GRM among AAW are not new, recent research has illuminated the particular forms of intersectional discrimination that AAW face, as well as the adverse mental health outcomes linked to GRM [7–11]. For instance, a multimethod qualitative study exploring the intersectional experiences of discrimination for AAW found six key themes of gendered racism that include experiences related to being (1) exoticized and fetishized (e.g., yellow fever), (2) not a leader, (3) submissive and passive, (4) cute and small, (5) invisible and silent, and (6) service workers [10]. Additionally, Keum and colleagues (2018) developed the Gendered Racial Microaggressions Scale for Asian American Women (GRMSAAW) which was comprised of four subscales: (a) ascribed submissiveness (i.e., AAW as always submissive and compliant), (b) assumption of universal appearance (i.e., all AAW share the same stereotypical appearance, e.g., petite with jet black hair), (c) Asian fetishism (i.e., exoticization of AAW), and (d) media invalidation (i.e., limited representation of AAW in media). Emerging evidence using the GRMSAAW shows that GRM experienced by Asian American women is linked to increased depressive symptoms, internalized racism, and suicide ideation [8, 9]. Given the increased mental health risks that AAW face, it is important to understand what factors may buffer or exacerbate the link between GRM perceptions and mental health outcomes. Thus, using a sample of AAW in romantic partnerships, the current study seeks to (a) examine the link between GRM and depressive symptoms among AAW in romantic partnerships and (b) assess whether partner race/ethnicity (White or Asian) may serve as a potential moderator in the relationship between GRM and depressive symptoms.

## The Role of Partner Race/Ethnicity

According to the resource model of marriage and partnership, partnered individuals tend to have greater access to emotional and material resources, which in turn contributes to better psychological well-being [12–14]. Partnered individuals tend to have more financial resources that can help reduce their exposure to stressors. Moreover, the exchange of support is perhaps most significant in improving health among partnered individuals. In particular, having a close companion and confidant that is responsive and able to listen to their problems and offer encouragement, care, and empathic responses can reduce stress and support better psychological well-being [12, 15, 16]. Indeed, a large body of empirical research demonstrates that partnered individuals are happier, less anxious, and less depressed than their single counterparts [12, 17–21].

However, union formation may not benefit all individuals equally. The homogamy hypothesis suggests that people tend to marry those who match with them on key status characteristics such as race/ethnicity, socioeconomic status, religion, and education [22]. Hence, dissimilar partners that are not aligned on key status characteristics may experience more conflicts that can contribute to increased depressive symptoms, psychological distress, and lower relationship quality that destabilizes the relationship and may eventually lead to separation [23–29]. For example, Bratter and King (2008) examined the likelihood of divorce among interracial couples compared to same-race couples and found that interracial couples, particularly White female/Black male and White female/Asian male marriages, were more prone to divorce compared to White/White couples. Similarly, Zhang and Van Hook (2009) found evidence that interracial marriages were less stable than endogamous marriages, with race or ethnicity showing strong associations with marital dissolution.

Along with being at greater risk for marital instability and dissolution, prior research has also indicated more adverse physical and mental health outcomes among individuals in interracial relationships. In a scoping review, individuals in interracial romantic relationships were found to have poorer physical health (e.g., low self-rated health) and psychological well-being, with several studies indicating greater depressive symptoms among individuals in interracial relationships compared to those in intraracial relationships [24]. Further, Pittman et al. (2023) found that compared to White couples, Black/White interracial couples were more likely to experience increased discrimination, perceived stress, depressive symptoms, and worse overall self-rated health. Additionally, associations between depressive symptoms and those in interracial relationships were partially mediated by discrimination and perceived stress among Black male/White female couples and fully mediated within Black women/White men couples.

Previous studies highlight differences in cultural upbringing, values, and relationship expectations (e.g., parenting style, conflict management, sexual scripts) may give rise to more conflict and eventually lead to separation among racially dissimilar partners [30–33]. In addition, experiences of discrimination and relationship disapproval from friends and family can also influence the health of interracial relationships, as well as both individuals [24, 34]. While these differences have been more frequently examined among Black/White couples, research has yet to demonstrate how differences in partner race/ethnicity among AAW in intraracial and interracial (Asian/White) partnerships shape experiences of discrimination and subsequent mental health outcomes.

## Asian-Asian Intraracial Couples and Gendered Racial Microaggressions

There is some evidence to suggest that Asian American women in intraracial partnerships may experience greater psychological well-being. Findings from a study examining the association between interracial relationships and depressive symptoms among a diverse sample of White, Black, Native American, Asian, and Hispanic respondents highlighted the psychological benefits associated with partnership formation (e.g., married, cohabiting). In particular, the evidence suggests that psychological benefits may only apply to those entering into intraracial relationships, with trends among Asian American women and men who entered into intraracial relationships showing a reduction in depressive symptoms [21]. Furthermore, a narrative analysis of U.S. born Asian Americans and their spousal preferences reveals important insights about how intraracial partnerships may generate important racial-ethnic relationship capital that can help buffer against perceived discrimination. Specifically, Asian Americans who preferred intraracial partnerships cite equality, racial empathy and understanding, enhanced rapport, and sharing a common culture as contributing to greater overall comfort [35]. The emphasis on sharing similar values and having a greater level of cultural comfort was also expressed in another study examining the meaning and dynamics of Asian American interethnic marriages [36]. Emerging literature among African American couples suggests that partnerships aligned on race/ethnicity may hold particular significance in countering the negative mental and physical health effects of discrimination through the receipt of racism-specific support provided by a partner who shares and understands the pain of experiencing racial discrimination [2].

However, there is also evidence to suggest that intraracial relationships may not be as helpful in buffering against racial discrimination. Results from a study examining the role of spousal support, discrimination, and psychological distress among Asian Americans show spousal support buffered against the negative effects of unfair treatment, but not racial discrimination [37]. Further, Chung and Epstein [38] found that in a sample of married or cohabiting foreign-born Asian immigrants, spousal support did not buffer the effects of racial discrimination on psychological distress. Rather, the association between discrimination and distress was heightened by spousal strain. Moreover, in a study of relationship outcomes among intraracial and interracial Asian-White couples, intraracial Asian couples were considered the least stable and had the least relationship satisfaction compared to the other racial pair groups (e.g., Asian male/White female, Asian female/White male, White male/White female; [39]). In a study investigating the health of intermarried adults, Asian American couples had worse self-rated health when compared to Asian American adults married to White spouses who reported the best health [40]. Indeed, being in an intraracial relationship does not guarantee greater psychological well-being and protection against the negative effects of racial discrimination. According to Pyke (2010) and Nemoto (2006), more traditional forms of Asian masculinity and ethnic patriarchy can still subject AAW to forms of GRM where they are expected to remain quiet, submissive, and compliant (e.g., ascribed submissiveness; [7]). Thus, contrary to what the homogamy hypothesis and prior research suggest, it remains unclear whether Asian American women in intraracial relationships are able to avoid the adverse mental health consequences associated with GRM.

## Asian-White Interracial Couples and Gendered Racial Microaggressions

Given AAW’s high intermarriage rates to White men and the particular salience of White hegemonic masculinity to GRM experiences such as Asian fetishism [7, 41–43], the current study examines AAW in interracial relationships with White male partners to further illuminate the role of partner race/ethnicity in shaping depressive symptoms among AAW exposed to GRM. Similar to AAW in intraracial relationships, there is research to suggest that AAW in interracial relationships with White men may experience some protection against the negative effects of GRM. Qualitative studies of AAW in interracial marriages with White men indicate a sort of protective privilege AAW feel they experience, where their White husband is able to shield them from discrimination and racial violence [44, 45]. For example, Greif et al. (2023) highlighted a few instances where AAW respondents felt vulnerable to racial animosity and violence (e.g., COVID-related anti-Asian attacks, being viewed as a terrorist), and being with their White husband conferred “life-protecting” privileges. Additionally, White male partners, especially those with a greater awareness of racism and White privilege, can be an important source of support and allyship against racial microaggressions from family members [44, 46]. For instance, in a study of the influence of interracial dating on the racial/ethnic identities of AAW and White men, one White male respondent described his role in challenging racial ignorance in his family and educating them about racism [46]. Taken together, these studies suggest that White male partners may help AAW mitigate potential harms associated with GRM through the protective privileges (i.e., power) they have to both challenge and shield against gendered racial oppression.

However, prior research has also indicated that instead of providing an important source of support against discrimination, White male partners can be the ones to perpetuate experiences of GRM for AAW [35, 41, 42, 45, 47]. Qualitative studies among AAW in interracial relationships with White men have highlighted similar themes of AAW often feeling like an exoticized other in their relationships with White men [35, 42, 45]. For example, Nemoto (2006) describes the emotional tensions and racial alienation that one Chinese American woman felt with her White boyfriend who was primarily attracted to and had only dated Asian girls. Specifically, her awareness of the ways that White men commodify and sexualize Asian women made her question whether it was her White boyfriend’s racist gaze or genuine love that kept them together [42]. Similarly, a qualitative study examining perceptions of societal microaggressions in Japanese American women married to White American men also identified the exoticization of Asian American women as a common experience. As one Japanese American woman recounted, simply being seen together with their White husbands was enough to elicit discrimination from a drunken soldier who called them “whores.” This form of sexual objectification was further illuminated by another respondent, who felt that many White men considered Asian women married to White men to be “bar girls” or “prostitutes” [45]. These studies underscore AAW’s increased vulnerability to GRM, particularly forms of sexual objectification and exoticization that may be more acutely felt by AAW in interracial relationships with White men. Yet, few studies examine the role of GRM in the context of Asian-White interracial relationships, and those that do utilize broad measures of discrimination that do not capture the unique intersecting oppressions that AAW face. Thus, further investigation is needed to understand the complex interplay between GRM, depressive symptoms, and partner race/ethnicity among AAW.

## The Present Study

According to our review of the literature, there is reason to believe that partner race/ethnicity, a proxy for disparate relational dynamics between interracial and intraracial relationships, may play a key role in moderating the link between GRM and depressive symptoms among AAW. Compared to individuals in intraracial relationships, research focused on mental health among individuals in interracial relationships demonstrates that they experience worse psychological well-being, with several studies indicating increased depressive symptoms [24, 27]. Prior studies suggest that AAW in intraracial relationships may report fewer depressive symptoms because there is a greater sense of equality, racial empathy, and cultural understanding, which may also facilitate more racism-specific support and mitigate harms related to GRM [2, 35]. Yet, depressive symptoms may also increase among AAW in intraracial relationships as they may encounter more instances of GRM (e.g., ascribed submissiveness) within the context of ethnic patriarchy [41, 42]. Conversely, AAW in interracial relationships, specifically with White men, may experience increased depressive symptoms if GRM are perpetuated by their partner directly, or if their partner minimizes or denies other GRM experiences [42, 45]. However, AAW in interracial relationships with White men may also experience some protective privileges that can help mitigate the risk for depressive symptoms linked to GRM [44, 45]. Thus, we aimed to examine the association between GRM and the severity of depressive symptoms and conducted a moderation analysis to assess how differences in partner race/ethnicity may strengthen or weaken this association. Below, we tested the following hypotheses:

- Hypothesis 1. Higher GRM stress will be significantly related to increased depressive symptoms among AAW.
- Hypothesis 2. Partner race/ethnicity status will moderate the link between GRM stress and depressive symptoms such that:
- (A) The relationship between GRM stress and depressive symptoms would be strengthened among AAW with White partners compared to AAW with Asian partners.
- (B) The relationship between GRM stress and depressive symptoms would be weakened or have a non-significant interaction effect among AAW with Asian partners compared to AAW with White partners.

## Method

### Participants

Data were collected from a sample of 156 AAW (*M*_age_ = 26.5, *SD* = 5.33). Most participants identified as women (99%) and one participant as genderfluid (0.6%). The sample was ethnically diverse and included Chinese (31%), Multiracial/Multiethnic (22%), Korean (12%), Vietnamese (10%), Indian (8%), Taiwanese (5%), Filipino (4%), and Bangladeshi (3%). The remaining 6% identified as Japanese, Cambodian, Thai, Hmong, Indonesian, Native Hawaiian or Pacific Islander, and other. Most participants identified as heterosexual (83%), and the remaining identified as bisexual (6%), uncertain (3%), other (2%), questioning (2%), queer (1%), gay (1%), lesbian (1%), and asexual (1%). About 69% identified as 2nd generation (born in the U.S. and at least one immigrant parent), 11% as 1st generation (born outside of the U.S.), 4% as 1.5 generation (immigrated to U.S. between ages 6–12), 4% as 1.75 generation (immigrated to U.S. between ages 0–5), 4% as adoptee, 3% as 1.25 generation (immigrated to U.S. between ages 13–17), 3% as 3rd generation (native born, at least both parents born in the U.S.), and 2% as other. More than half of the participants identified their partner’s race/ethnicity as White (53%) and 47% as Asian.

### Procedure

The current study used a subset data from IRB-approved data collected previously by Keum et al. (2018). Participants were invited to complete an online survey hosted by Qualtrics, consisting of study variable measures, demographic items, and two attention check items (e.g., “Please choose always”). Participants were recruited through online platforms catering to Asian women residing in the U.S. (e.g., Facebook pages, Google groups), e-mail (e.g., listservs), and discussion forums. The inclusion criteria for participants to partake in the study were (1) 18 years old or older; (2) self-identify as Asian/Asian American women; and (3) live in the USA. Informed consent was provided and obtained from all participants. Participants completed the survey within 15–20 min.

### Measures

#### Gendered Racial Microaggression Stress

Gendered Racial Microaggressions Scale for Asian American Women (GRMSAAW; [7]) is a 22-item, four-factor scale that assesses the behavioral, verbal, and environmental manifestations of gendered racial microaggressions experienced by AAW in the United States. The four subscales are (a) ascribed submissiveness (AS; nine items); (b) assumption of universal appearance (AUA; four items); (c) Asian fetishism (AF; four items); and (d) media invalidation (MI; five items). We asked participants to report the stressfulness of their experiences on the Gendered Racial Microaggressions Stress (GRMS) scale that utilizes a six-point Likert scale (0 = *not at all stressful* to 5 = *extremely stressful*). A sample item includes “Others treat me as if I will always comply with their requests,” or “Others have talked about Asian American women as if they all have the same facial features.” Higher scores indicate greater gendered racial microaggression stress. Internal consistency estimates were 0.80 and above for frequency. Construct validity was established as GRMSAAW scores were associated with racial microaggressions, sexism, depressive symptoms, and internalized racism [7]. Cronbach’s alphas for the current study were 0.91, 0.88, 0.90, and 0.89 for AS, AF, AUA, and MI, respectively.

#### Depressive Symptoms

The Patient Health Questionnaire-9, a nine-item depression scale, was used to measure and establish provisional depressive disorder diagnoses and the severity of depressive symptoms. (PHQ-9; [48]). The PHQ-9 assesses how often participants have been bothered by the following over the past 2 weeks. Sample items include the following: “Little interest or pleasure in doing things?”; “Feeling down, depressed, or hopeless?”; “Trouble falling or staying asleep, or sleeping too much.” Scores are summed up and higher scores indicate increasing severity for depressive symptomatology. Scores on the PHQ-9 were correlated with the Symptom Checklist-20 [48]. Validity and measurement invariance of PHQ-9 with Asian American college students has been supported [7]. Cronbach’s alpha for the current study was 0.90.

### Data Analysis

The data were analyzed using SPSS 24 [49]. First, bivariate correlations and descriptive statistics of the study variables were assessed. Covariates including socioeconomic status, sexual orientation, nativity, generational status, and ethnic background have been found to be associated with both racial discrimination and mental health outcomes among Asian American samples and were included in all of our analyses [50–53]. For instance, compared to heterosexual AAW, GRM may be more distressing for sexual minority AAW who experience fetishization for their race as well as their sexuality (e.g., lesbian fetishization; [53]). Additionally, awareness and perception of GRM may vary based on nativity and generational status, where AAW from later generations (e.g., 2nd, 3rd generation) may experience greater internalized racism and are less aware of GRM than earlier generations (e.g., 1st generation) AAW [7, 50]. We first examined the main effects of the GRMS subscales on depressive symptoms (PHQ-9) by conducting multiple regressions. To test whether partner race/ethnicity (Asian or White) moderates these main effects, we conducted conditional process modeling [49]. We selected and ran model 1 in process macro version 3.5 for SPSS Hayes (2018) to conduct a moderated linear regression between GRMS subscales (IV) and depressive symptoms as the outcome (DV), and partner race/ethnicity as the moderator (W), with bias-corrected bootstrapping (10,000 resamples). PROCESS uses an ordinary least squares regression-based path analytic framework for estimating the interactions in moderation models along with simple slopes for probing the interactions. Significance of the simple slopes was assessed if the interaction was significant. We used Ferguson’s [54] criteria to assess effect size for the strength of association indices such as *r* and path coefficients, in which 0.20 is the recommended minimum effect size representing a practically significant effect, 0.50 represents a moderate effect and 0.80 represents a large effect.

## Results

### Data Inspection and Preliminary Analysis

A total of 156 individuals participated in the survey. There were no missing data among those who provided partner race/ethnic background information. Descriptive and bivariate correlations of study variables are listed in Table 1. Bivariate correlations suggested that depressive symptoms were significantly correlated with the GRMS subscales for ascribed submissiveness, Asian fetishism, and media invalidation at small effect. Using the criteria that kurtosis and skewness are between − 2 and + 2, all variables were considered to have normal distribution [55]. All VIF values were less than 2 (range 1.61 to 1.91), suggesting little to no multicollinearity [55]. Regression assumptions for linearity and normality and little to no multicollinearity were satisfied.

**Table 1LE: Please check if the table captions, table entries, table note, and other relevant details in Tables 1 and 2 are presented correctly. Otherwise, please amend.** Descriptive statistics and bivariate correlations of study variables

Variables	Correlation					Descriptive						
	1	2	3	4	5	Min	Max	*M*	*SD*	Skewness	Kurtosis	*⍺*
1. AS	-					1.00	6.00	3.41	1.32	− .04	− .92	.91
2. AF	.67**	-				1.00	6.00	3.66	1.53	− .16	− 1.20	.88
3. MI	.58**	.69**	-			1.00	6.00	4.38	1.38	− .80	− .41	.89
4. AUA	.57**	.60**	.69**	-		1.00	6.00	4.09	1.21	− .56	− .37	.90
5. PHQ-9	.31**	.23**	.21*	0.11	-	9.00	34.00	15.96	5.72	1.12	.87	.90

*Note. AS* ascribed submissiveness, *AF* Asian fetishism, *MI* media invalidation, *AUA* assumptions of universal appearance, *PHQ-9* Patient Health Questionnaire-9

^**^p < .01; *p < .05

### Moderation Model for Gendered Racial Microaggressions

To assess the main effects of GRMS on depressive symptoms among a sample of AAW, regression analysis was conducted. Multiple regression results suggest that when sociodemographic characteristics (e.g., socioeconomic status, sexual orientation, nativity, generational status, and ethnic background) are taken into account, the association between GRMS and depressive symptoms is significant for all subscale factors. Specifically, it was found that ascribed submissiveness (*β* = 0.30, *p* < .001), Asian fetishism (*β* = 0.23, *p* < .001), assumptions of universal appearance (*β* = 0.15, *p* < .01), and media invalidation (*β* = 0.18, *p* < .05) all significantly predicted depressive symptoms.

**Fig. 1:**
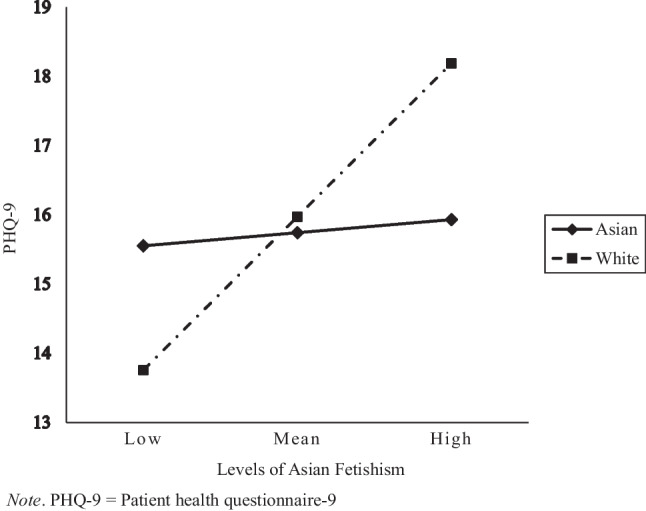
Partner race/ethnicity as a moderator between Asian fetishism and depressive symptoms

Results from the PROCESS macro analysis with bias-corrected bootstrapping (10,000 resamples) revealed that partner race/ethnicity did not moderate the association between GRMS subscales of ascribed submissiveness, assumptions of universal appearance, and media invalidation with depressive symptoms. However, partner race/ethnicity did moderate the association between Asian fetishism and depressive symptoms (*F*(1, 145) = 4.82, *p* = .03, Δ*R*^2^ = 0.03). Table 2 presents the estimates of the moderated regression model for Asian fetishism and depressive symptoms. Simple slopes at mean levels of Asian fetishism among White partners and Asian partners were assessed to support the interpretation of the significant interaction (Fig. 1). Specifically, for AAW with White partners, slopes were positive and significant (*t* = 3.452, *β* = 1.45, *SE* = 0.42, *p* < .001), suggesting that experiences of Asian fetishism were significantly associated with depressive symptoms for AAW. However, for AAW with Asian partners, slopes were positive but not significant (*t* = 0.285, *β* = 0.12, *SE* = 0.43, *p* = .776), suggesting that the association between Asian fetishism and depressive symptoms varies depending on partner race/ethnicity.

**Table 2:** Bootstrapped estimate of interaction between Asian fetishism and partner race/ethnicity predicting depressive symptoms

Variables	*β*	*SE*	*t*	*p*-value	CI
AF	.12	.43	.29	0.776	[− .73, .98]
Partner race/ethnicity	.23	.94	.25	0.806	[− 1.62, 2.08]
AF × partner race/ethnicity	1.32	.60	2.20	0.03	[.13, 2.51]

*Note. AF* Asian fetishism; Partner race/ethnicity reference group = Asian; *CI* confidence intervals. Model adjusts for sexual orientation, nativity, race/ethnicity, socioeconomic status, and generational status

## Discussion

Prior studies have highlighted the important role of romantic partners in reducing discrimination-related stress and improving psychological well-being [2]. However, few studies have investigated how the relational context can shape depressive symptoms among AAW that encounter GRM. To address this gap, we sought to explore whether partner race/ethnicity (i.e., White and Asian partner) would moderate the link between GRM stress and depressive symptoms among AAW. Greater perceived GRM stress (i.e., ascribed submissiveness, assumption of universal appearance, Asian fetishism, and media invalidation) was significantly associated with more severe depressive symptoms among AAW. However, the associations between GRM dimensions of ascribed submissiveness, assumption of universal appearance, and media validation with depressive symptoms were not conditional on partner race/ethnicity. Only Asian fetishism-related GRM stress and depressive symptoms among AAW were moderated by partner race/ethnicity. The lack of significant moderation between the other dimensions of GRM with partner race/ethnicity does not diminish the significance of the moderating role of partner race/ethnicity. Rather, our findings draw attention to the particular salience of Asian fetishism-related GRM among AAW with White partners. In line with prior research that suggests that GRM may feel more pronounced for AAW with White male partners [35, 41, 42, 45, 47], experiences of Asian fetishism were significantly and positively associated with increased depressive symptoms for AAW with White partners. Conversely, experiences of Asian fetishism among AAW with Asian male partners were positive, but the association was non-significant.

According to our findings, increased exposure to Asian fetishism-related GRM may be especially detrimental for AAW with White partners who reported greater depressive symptoms. It seems our findings may be consistent with prior research that indicates greater depressive symptoms among individuals in interracial relationships compared to those in intraracial relationships [24]. Additionally, our findings add to the growing body of research examining the role of discrimination on mental and physical well-being in interracial and intraracial couples [2, 27, 56]. Specifically, we identify GRM as a unique form of discrimination among AAW in interracial relationships with White men that can contribute to adverse mental health outcomes, such as increased depressive symptoms. In line with previous studies, it is possible that White male partners perpetuate GRM directly to their Asian American female partner [35, 41, 42, 45, 47]. The impact of GRM from White male partners may be especially distressing given the deep-seated history of sexual objectification and exoticization of Asian women in the U.S. (e.g., mail-order brides, war brides, sex tourism, pornography), which has been further cemented into popular culture through films and other media (e.g., Dragon Lady, Lotus Blossom; [57]). Additionally, Nemoto (2006) highlights that these stereotypical depictions of Asian women are often constructed in relation to White men, solidifying their superordinate position in the racial/gender hierarchy and AAW’s subordinate position. For the AAW in our sample, the majority of whom are 2nd generation and likely have more socialization around various forms of U.S.-based racism including GRM [42, 58], Asian fetishism may evoke a sense of marginalization and subordination that contributes to greater depressive symptoms for AAW with White partners.

It is also possible that White male partners are not the ones to directly perpetuate GRM but may provide inadequate support for their AAW partner. Instead of validating or affirming AAW’s GRM experiences, they may minimize, deny, silence, or even victim blame their AAW partner for the GRM they encounter [59]. For example, instead of being supportive, White male partners may tell AAW that they are being overly sensitive, or even blame their AAW partner for the sexual objectification they receive. Indeed, previous studies have indicated that while Japanese American women reported their White husbands to be generally supportive, there were a few participants whose White husbands minimized their experience of discrimination and microaggressions, telling them not to make “a big scene, it’s nothing” [45]. Relatedly, the sense of support that AAW feel they have may also extend to their social context. For instance, AAW with White male partners may live in settings where they are more exposed to GRM (e.g., predominantly White social functions, White cultural dynamics, White in-laws and extended family; [42, 44, 45]). Thus, our findings help extend the homogamy hypothesis, showing that it is salient for future research to consider the role of intersectional forms of discrimination, such as GRM, to contextualize why race/ethnicity is a significant status characteristic in shaping AAW well-being in interracial relationships with White men.

Conversely, for AAW with Asian male partners, there was a non-significant association between Asian fetishism-related GRM and depressive symptoms. Our findings are consistent with studies that support the homogamy hypothesis, which suggests that individuals in intraracial relationships (i.e., matching on race/ethnicity) report diminished conflict and greater psychological well-being than dissimilar partners (i.e., interracial couples) [21, 26, 29]. As other studies have indicated, having a shared cultural background, racial empathy, and greater sense of equality can help AAW feel more overall comfort [35]. In particular, it is possible that having shared cultural values, traditions, and family expectations may facilitate a greater sense of connection and understanding that goes beyond gendered racial stereotypes, to recognize AAW’s individuality and autonomy. Instead of seeing AAW as one-dimensional exoticized “others,” [42], Asian male partners may be more likely to draw from their own relationships with the multifaceted AAW in their lives (e.g., grandmother, mother, sister) and view their AAW partner as a complex individual with unique needs and interests.

Additionally, in instances where AAW encounter Asian fetishism from others, Asian male partners may be uniquely positioned to offer racism-specific social support [2]. Beyond more general forms of social support, racism-specific support provides a safe and intimate space to vent about encounters with racial discrimination with a partner who can validate, empathize, and share a mutual understanding of the pain from witnessing or experiencing racial discrimination firsthand [1]. Moreover, it may be especially therapeutic for AAW to confide in Asian male partners who are critically aware of their own experiences of GRM (e.g., sexualized emasculation) [60]. Rather than taking the time to explain and educate their partner about why GRM are offensive and harmful, they can engage more deeply in reflective dialogue that is affirming and allows them to challenge Asian fetishism stereotypes and oppressive ideologies together [61]. Finally, it is also possible that AAW in intraracial relationship may live in social contexts where there is limited exposure to Asian fetishization. For instance, in a study of ethnic enclaves, discrimination, and stress among Asian American women, [62] found AAW living in neighborhoods with more cultural institutions (e.g., Asian serving religious, social, civic organizations) reported lower discrimination. Thus, Asian American intraracial couples may live in areas where there is a greater density of Asian Americans and more access to Asian American serving institutions and resources that provide spaces that can help limit AAW’s encounters with GRM.

Nevertheless, it is important to exercise caution when interpreting these findings. The data do not suggest that all AAW with White partners are at an increased risk for worse mental health outcomes related to GRM. Rather, the strength of the association between GRM and depressive symptoms for AAW may depend upon their partners’ level of critical consciousness, as well as their own. Critical consciousness can support an individual’s ability to resist discrimination by critically reflecting, challenging, and being motivated to take action against social inequities [63–65]. Recent research examining Asian/White interracial heterosexual couples’ psychological distress and relationship satisfaction highlights greater psychological distress and lower relationship satisfaction among Asian partners with higher critical consciousness that perceive more frequent blatant racism [65]. However, the positive associations between perceived blatant or subtle racism and psychological distress were buffered by their White partners’ critical consciousness, as they were more equipped to empathize and support their Asian female partners in coping with the negative impact of racism [65]. Thus, it is important to develop greater critical consciousness among AAW and their romantic partners of any race/ethnicity, including AAW in intraracial relationships, as Asian male partners may also perpetuate GRM or provide inadequate support around GRM-related incidents.

### Limitations and Future Directions

Despite noteworthy findings, the study has some limitations. First, the use of cross-sectional data restricts our ability to establish the temporal sequence of our study variables, limiting any causal implications. Longitudinal data would allow for future studies to replicate our findings and provide insights into how partner race/ethnicity moderates the association between GRM and depressive symptoms over time. Second, while GRM related to Asian fetishism has been reported among South and Southeast Asian American women as well as East Asian women [41, 42, 66], not all GRM (e.g., assumptions of universal appearance; petite frame, porcelain skin) may be shared across different Asian ethnicities [10, 52]. Thus, our study is limited in generalizability to the major identities represented in our sample (East Asian, heterosexual, 2nd generation AAW). Future research should incorporate a more representative sample that includes larger samples of South and Southeast Asian American women as well as AAW across diverse age groups, generational statuses, and sexual orientations. Third, our findings would need to be extended with AAW in relationships with partners of other racial and ethnic backgrounds (e.g., Black American, Latino, Middle Eastern) and sexual orientations. Greif et al. (2023) highlight differing dynamics among Asian-Black couples, where AAW recognize the racial status of their Black male partner may elicit increased racial discrimination for both parties [44]. Additionally, prior studies have indicated greater distress among LGBTQ + AAW who experience fetishization because of their race, gender, and sexuality [53]. However, it is unclear how perceptions of GRM might differ for LGBTQ + AAW in relationships with other racial and sexual minorities. Thus, future studies would benefit from extending our results with romantic partners of diverse races/ethnicities and sexual orientations. Fourth, while our study provided valuable insights into the moderating role of partner race/ethnicity in the link between GRM and depressive symptoms among AAW, we are unable to draw any conclusions about the role of partner support and relationship quality in shaping experiences of GRM. Future studies would benefit from directly examining these relational processes alongside experiences of GRM to understand important nuances in support and relationship dynamics that can influence mental health outcomes.

Finally, although the study employed an innovative measure (GRMSAAW; [7]) to capture GRM experiences among AAW, we are unable to determine the source of the GRM. Having the source of the GRM can provide further specificity (e.g., whether their GRM experiences stem from partners, family, friends, or broader social context) regarding the role of partner race/ethnicity in shaping perceptions of GRM. AAW may be in supportive relationships but face increased stress and stigma from their community for choosing to date outside of their own racial/ethnic group, particularly AAW that decide to date White men. For instance, AAW that date White men have experienced an explosive growth in digital intimidation tactics (e.g., harassment, hate speech, slurs, rape, death threats) by the MRAsians movement, an anti-feminist Asian American men’s rights subculture that views Asian women in relationships with non-Asian men as “self-hating” and “white worshipping,” often calling AAW “race traitors” [67, 68]. These experiences of harassment and hate may be especially stressful for AAW. In particular, their choice to date White men could be viewed as their willful reproduction of the stereotype of Asian fetishism, ultimately reduced to yet another “WMAF” or “White Male/Asian Female” couple. Thus, future studies would benefit from the examination of other sources of GRM outside of AAW’s immediate romantic partnerships which may worsen their mental health outcomes.

### Implications for Practice and Policy

Our findings have important implications for interventions that practitioners can employ to help AAW mitigate the harmful effects of GRM that may occur within their relationships with romantic partners of different racial/ethnic backgrounds. From a micro-level, human service providers can utilize interventions such as narrative therapy or acceptance-based psychotherapies to help AAW identify their strengths, take control of their story, and develop effective coping mechanisms in managing their symptoms of depression and the impacts of GRM [69]. Practitioners can also integrate the development of greater critical consciousness to empower AAW to challenge stereotypes and increase their partners’ awareness and understanding of what GRM are and how they can better support their AAW partner when they are exposed to GRM. Further, practitioners can assist White male partners in developing greater awareness around their power and privilege and how they can use their position to help educate others (e.g., friends, family members) that may perpetuate GRM [46].

Furthermore, public and private institutions at mezzo- and macro-levels can develop a broader range of public services that are tailored to the diverse needs of AAW experiencing GRM from their romantic partners, particularly among AAW who experience increased violence and aggression related to GRM. For example, schools can incorporate a more comprehensive sex education program that utilizes an intersectional approach in the development of healthy relationships, communication skills, and preventing dating violence [70]. In particular, instructors can apply a rights-based approach to increase awareness of how GRM can manifest and limit AAW’s fundamental rights to nondiscrimination [71]. Additionally, policymakers can work to create an environment that protects AAW by supporting media literacy campaigns that aim to dismantle “yellow fever” narratives that objectify AAW [57, 72]. Policymakers can also help establish community-based organizations that work with administrators and executives within the entertainment and media industries to regulate the production of media content to eliminate the reproduction of harmful images and stereotypes of AAW.

### Conclusion

In conclusion, this article illuminates the role of partner race/ethnicity as a major factor that can moderate the link between Asian fetishism-related GRM stress and depressive symptoms among AAW. Specifically, experiences of Asian fetishism, a dimension of GRM stress, may feel uniquely oppressive for AAW with White male partners and lead to increased reports of depressive symptoms, whereas the link between GRM and depressive symptoms is no longer significant among AAW with Asian male partners. These findings contribute further evidence to growing research on the adverse mental health outcomes related to GRM for AAW [8–10, 51] and identified partner race/ethnicity as a significant factor that can help contextualize the harmful consequences of GRM. Future research should explore other factors such as internalized racism and color-blind racial attitudes that can further contextualize the relationship between GRM stress and depressive symptoms among AAW in intraracial and interracial partnerships [50, 51]. Additionally, mental health providers and practitioners should utilize an intersectional approach to identify GRM and other intersecting oppressions among AAW clients that may increase their risk for adverse mental health outcomes. Together, this research lays the foundation for future policies and interventions to strengthen support for AAW through comprehensive educational programs and media literacy campaigns that have the potential to dismantle harmful stereotypes of AAW and minimize GRM within their social context.

## Data Availability

Data used in this study may be available upon request.

## Code Availability

Code used in this study may be available upon request.

## References

[1] Donnelly R, Robinson BA, Umberson D. Can spouses buffer the impact of discrimination on depressive symptoms? An examination of same-sex and different-sex marriages. Soc Mental Health. 2019;9(2):192–210. 10.1177/2156869318800157 PMC6585990 31223514

[2] McNeil Smith S, Williamson LD, Branch H, Fincham FD. Racial discrimination, racism-specific support, and self-reported health among African American couples. J Soc Pers Relat. 2020;37(3):779–99.

[3] McNeil SN, Fincham FD, Beach SRH. Does spousal support moderate the association between perceived racial discrimination and depressive symptoms among African American couples? Fam Process. 2014;53(1):109–19. 24251910 10.1111/famp.12054

[4] Crenshaw K. Demarginalizing the intersection of race and sex: a black feminist critique of antidiscrimination doctrine, feminist theory and antiracist politics. U Chi Legal F. 1989;139.

[5] Essed P. Understanding everyday racism: an interdisciplinary theory. SAGE; 1991. 333 p.

[6] Lewis JA, Neville HA. Construction and initial validation of the Gendered Racial Microaggressions Scale for Black women. J Couns Psychol. 2015;62(2):289–302. 25867696 10.1037/cou0000062

[7] Keum BT, Brady JL, Sharma R, Lu Y, Kim YH, Thai CJ. Gendered Racial Microaggressions Scale for Asian American women: development and initial validation. Journal of counseling psychology. 2018. 10.1037/cou0000305 30058827

[8] Keum BT, Wong MJ, Salim-Eissa R. Gendered racial microaggressions, internalized racism, and suicidal ideation among emerging adult Asian American women. International Journal of Social Psychiatry. 2022. 10.1177/00207640221089536 PMC9983054 35411802

[9] Keum BT, Wong M. Congruence and discrepancy in Asian American women’s perception and stress appraisal of gendered racial microaggressions: relationships with depressive symptoms and internalized racism. Frontiers in Public Health [Internet]. 2022 [cited 2022 Oct 13];10. Available from: https://www.frontiersin.org/articles/10.3389/fpubh.2022.954897 10.3389/fpubh.2022.954897 PMC9641222 36388393

[10] Mukkamala S, Suyemoto KL. Racialized sexism/sexualized racism: a multimethod study of intersectional experiences of discrimination for Asian American women. Asian Am J Psychol. 2018;9(1):32.

[11] Forbes N, Yang LC, Lim S. Intersectional discrimination and its impact on Asian American women’s mental health: a mixed-methods scoping review. Frontiers in Public Health [Internet]. 2023 [cited 2023 Dec 10];11. Available from: https://www.frontiersin.org/articles/10.3389/fpubh.2023.993396 10.3389/fpubh.2023.993396 PMC10008964 36923035

[12] Kiecolt-Glaser JK, Wilson SJ. Lovesick: how couples’ relationships influence health. Annu Rev Clin Psychol. 2017;13(1):421–43. 28301763 10.1146/annurev-clinpsy-032816-045111 PMC5549103

[13] Umberson D, Thomeer MB, Williams K. Family status and mental health: recent advances and future directions. In: Aneshensel CS, Phelan JC, Bierman A, editors. Handbook of the Sociology of Mental Health [Internet]. Dordrecht: Springer Netherlands; 2013 [cited 2023 Dec 10]. p. 405–31. (Handbooks of Sociology and Social Research). Available from: 10.1007/978-94-007-4276-5_20

[14] Waite L, Gallagher M. The case for marriage: why married people are happier, healthier and better off financially. Crown; 2001. 274 p.

[15] Meuwly N, Bodenmann G, Germann J, Bradbury TN, Ditzen B, Heinrichs M. Dyadic coping, insecure attachment, and cortisol stress recovery following experimentally induced stress. J Fam Psychol. 2012;26(6):937–47. 23127351 10.1037/a0030356

[16] Thoits PA. Mechanisms linking social ties and support to physical and mental health. J Health Soc Behav. 2011;52(2):145–61. 21673143 10.1177/0022146510395592

[17] Braithwaite SR, Delevi R, Fincham FD. Romantic relationships and the physical and mental health of college students. Pers Relat. 2010;17(1):1–12.

[18] DeKlyen M, Brooks-Gunn J, McLanahan S, Knab J. The mental health of married, cohabiting, and non–coresident parents with infants. Am J Public Health. 2006;96(10):1836–41. 16571717 10.2105/AJPH.2004.049296 PMC1586128

[19] Demir M. Close relationships and happiness among emerging adults. J Happiness Stud. 2010;11(3):293–313.

[20] Musick K, Bumpass L. Reexamining the case for marriage: union formation and changes in well-being. J Marriage Fam. 2012;74(1):1–18. 22611285 10.1111/j.1741-3737.2011.00873.x PMC3352182

[21] Wong JS, Penner AM. Better together? Interracial relationships and depressive symptoms. Socius. 2018;1(4):2378023118814610.

[22] Kalmijn M. Intermarriage and homogamy: causes, patterns, trends. Ann Rev Sociol. 1998;24(1):395–421. 10.1146/annurev.soc.24.1.395 12321971

[23] Bratter JL, King RB. “But will it last?”: marital instability among interracial and same-race couples*. Fam Relat. 2008;57(2):160–71.

[24] Calderon PSP, Wong JD, Hodgdon BT. A scoping review of the physical health and psychological well-being of individuals in interracial romantic relationships. Fam Relat. 2022;71(5):2011–29.

[25] Hohmann-Marriott BE, Amato P. Relationship quality in interethnic marriages and cohabitations. Soc Forces. 2008;87(2):825–55.

[26] Joyner K, Kao G. Interracial relationships and the transition to adulthood. Am Sociol Rev. 2005;70(4):563–81.

[27] Pittman PS, Kamp Dush C, Pratt KJ, Wong JD. Interracial couples at risk: discrimination, well-being, and health. Journal of Family Issues. 2023 Jan 12;0192513X221150994. 10.1177/0192513x221150994 PMC12346691 40809512

[28] Schwartz CR. Trends and variation in assortative mating: causes and consequences. Ann Rev Sociol. 2013;39(1):451–70.

[29] Zhang Y, Van Hook J. Marital dissolution among interracial couples. J Marriage Fam. 2009;71(1):95–107. 25284887 10.1111/j.1741-3737.2008.00582.x PMC4183451

[30] Crippen C, Brew L. Strategies of cultural adaption in intercultural parenting. Fam J. 2013;21(3):263–71.

[31] Gottman JM. A theory of marital dissolution and stability. J Fam Psychol. 1993;7(1):57–75.

[32] Gurung RAR, Duong T. Mixing and matching: assessing the concomitants of mixed-ethnic relationships. J Soc Pers Relat. 1999;16(5):639–57.

[33] Rowley CA, Craft AL, Perry-Jenkins M. Parental conflict in the context of multiethnoracial relationships. J Child Fam Stud. 2022;31(3):649–63. 36213085 10.1007/s10826-022-02249-6 PMC9544355

[34] LeBlanc AJ, Frost DM, Wight RG. Minority stress and stress proliferation among same-sex and other marginalized couples. J Marriage Fam. 2015;77(1):40–59. 25663713 10.1111/jomf.12160 PMC4316376

[35] Chow S. The significance of race in the private sphere: Asian Americans and spousal preferences. Sociol Inq. 2000;70(1):1–29.

[36] Chong KH. “Asianness” under construction: the contours and negotiation of panethnic identity/culture among interethnically married Asian Americans. Sociol Perspect. 2017;60(1):52–76.

[37] Rollock D, Lui PP. Do spouses matter? Discrimination, social support, and psychological distress among Asian Americans. Cultural Diversity and Ethnic Minority Psychology. 2016 Jan;22(1):47–57. 10.1037/cdp0000045 25867552

[38] Chung H, Epstein NB. Perceived racial discrimination, acculturative stress, and psychological distress among asian immigrants: the moderating effects of support and interpersonal strain from a partner. Int J Intercult Relat. 2014;1(42):129–39.

[39] Canlas JM, Miller RB, Busby DM, Carroll JS. Same-race and interracial Asian-White couples: relational and social contexts and relationship outcomes. J Comp Fam Stud. 2015;46(3):307–28.

[40] Miller B, Kail BL. Exploring the effects of spousal race on the self-rated health of intermarried adults. Sociol Perspect. 2016;59(3):604–18.

[41] Pyke K. An intersectional approach to resistance and complicity: the case of racialised desire among Asian American women. J Intercult Stud. 2010;31(1):81–94.

[42] Nemoto K. Intimacy, desire, and the construction of self in relationships between Asian American women and White American men. Journal of Asian American Studies. 2006;9(1):27–54.

[43] Livingston G, Brown A. 1. Trends and patterns in intermarriage [Internet]. Pew Research Center’s Social & Demographic Trends Project. 2017 [cited 2022 Jan 26]. Available from: https://www.pewresearch.org/social-trends/2017/05/18/1-trends-and-patterns-in-intermarriage/

[44] Greif GL, Chung Y, Lee H, Zhang P. The experiences of Asian Americans who intermarry in the United States: a qualitative study. Fam Soc. 2023;6:10443894231193376.

[45] Iwasaki M, Thai CJ, Lyons HZ. Perceptions of societal microaggressions in Japanese American women married to White American men. Couple Fam Psychol Res Pract. 2016;5(3):180–96.

[46] AhnAllen JM, Suyemoto KL. Influence of interracial dating on racial and/or ethnic identities of Asian American women and white European American men. Asian Am J Psychol. 2011;2(1):61–75.

[47] Kim B. Asian female and Caucasian male couples: exploring the attraction. Pastoral Psychol. 2011;60(2):233–44.

[48] Kroenke K, Spitzer RL. The PHQ-9: a new depression diagnostic and severity measure. Psychiatr Ann. 2002;32(9):509–15.

[49] Hayes AF. The PROCESS macro for SPSS and SAS (Version 3.5) [Internet]. 2018. Available from: http://www.processmacro.org/download.html

[50] Choi AY, Israel T, Maeda H. Development and evaluation of the Internalized Racism in Asian Americans Scale (IRAAS). J Couns Psychol. 2017;64(1):52. 28068131 10.1037/cou0000183

[51] Keum BT, Miller MJ, Lee M, Chen GA. Color-Blind Racial Attitudes Scale for Asian Americans: testing the factor structure and measurement invariance across generational status. Asian Am J Psychol. 2018;9(2):149–57.

[52] Poolokasingham G, Spanierman L, Kleiman S, Houshmand S. “Fresh off the boat?” Racial microaggressions that target South Asian Canadian students. J Divers High Educ. 2014;1(7):194–210.

[53] Sung MR, Szymanski DM, Henrichs-Beck C. Challenges, coping, and benefits of being an Asian American lesbian or bisexual woman. Psychol Sex Orientat Gend Divers. 2015;2(1):52–64.

[54] Ferguson CJ. An effect size primer: a guide for clinicians and researchers. Washington, DC, US: American Psychological Association; 2016. 301 p. (Methodological issues and strategies in clinical research, 4th ed).

[55] George D, Mallery P. SPSS for windows step by step: a simple study guide and reference 17.0 update. Boston, MA: Pearson Education, Inc.; 2010.

[56] Irby-Shasanmi A, Erving CL. Do discrimination and negative interactions with family explain the relationship between interracial relationship status and mental disorder? Socius. 2022;1(8):23780231221124852. 10.1177/23780231221124852 PMC9601714 36303609

[57] Zheng R. Why yellow fever isn’t flattering: a case against racial fetishes. J of the Am Philos Assoc. 2016;2(3):400–19.

[58] Ahn LH, Keum BT, Meizys GM, Choudry A, Gomes MA, Wang L. Second-generation Asian American women’s gendered racial socialization. J Couns Psychol. 2022;69(2):129–45. 34242043 10.1037/cou0000575

[59] Wang SC, Santos BMC. “What support?”: a qualitative study on social support for Asian American victims of racism during the COVID-19 pandemic. Frontiers in Public Health [Internet]. 2022 [cited 2023 Nov 29];10. Available from: https://www.frontiersin.org/articles/10.3389/fpubh.2022.961215 10.3389/fpubh.2022.961215 PMC9626990 36339164

[60] Keum BT, Ahn LH, Choi AY, Choudhry A, Nguyen M, Meizys GM, et al. Asian American men’s gendered racial socialization and fragmented masculinity: interpretive phenomenological analysis. Couns Psychol. 2023;51(5):684–718.

[61] Gockel A, O’Neill P, Pole N. Social justice conversations: using critical dialogue to unpack oppression. Fam Soc. 2022;103(4):377–93.

[62] Morey BN, Link to external site this link will open in a new window, Gee GC, Link to external site this link will open in a new window, Shariff-Marco S, Yang J, et al. Ethnic enclaves, discrimination, and stress among Asian American women: differences by nativity and time in the United States. Cultural Diversity and Ethnic Minority Psychology. 2020 Oct;26(4):460–71. 10.1037/cdp0000322 32091229

[63] Diemer MA, Kauffman A, Koenig N, Trahan E, Hsieh CA. Challenging racism, sexism, and social injustice: support for urban adolescents’ critical consciousness development. Cultur Divers Ethnic Minor Psychol. 2006;12(3):444–60. 16881749 10.1037/1099-9809.12.3.444

[64] Freire P. Pedagogy of the oppressed. 30th ed. Continuum; 2020.

[65] Tao C. The role of critical consciousness on Asian-White interracial couples’ perceived racism and well-being: a mixed-methods study [Internet] [Ph.D.]. [United States -- Arizona]: Arizona State University; 2020 [cited 2021 Nov 16]. Available from: https://www.proquest.com/docview/2436879332/abstract/AED95D1F27754ABAPQ/1

[66] Patel NR. The construction of South-Asian-American womanhood. Women Ther. 2007;30(3–4):51–61.

[67] Liu A. MRAsians: a convergence between Asian American hypermasculine ethnonationalism and the manosphere. J Asian Am Stud. 2021;24(1):93–112.

[68] Ng C. When Asian women are harassed for marrying non-Asian men [Internet]. The Cut. 2018 [cited 2022 Sep 18]. Available from: https://www.thecut.com/2018/10/when-asian-women-are-harassed-for-marrying-non-asian-men.html

[69] Hall GCN, Hong JJ, Zane NWS, Meyer OL. Culturally competent treatments for Asian Americans: the relevance of mindfulness and acceptance-based psychotherapies. Clin Psychol Sci Pract. 2011;18(3):215–31. 10.1111/j.1468-2850.2011.01253.x PMC3208524 22065893

[70] Goldfarb ES, Lieberman LD. Three decades of research: the case for comprehensive sex education. J Adolesc Health. 2021;68(1):13–27. 33059958 10.1016/j.jadohealth.2020.07.036

[71] Rohrbach LA, Berglas NF, Jerman P, Angulo-Olaiz F, Chou CP, Constantine NA. A rights-based sexuality education curriculum for adolescents: 1-year outcomes from a cluster-randomized trial. J Adolesc Health. 2015;57(4):399–406. 26403840 10.1016/j.jadohealth.2015.07.004

[72] Sun C, Liberman R, Butler A, Lee SY, Webb R. Shifting receptions: Asian American stereotypes and the exploration of comprehensive media literacy. Commun Rev. 2015;18(4):294–314.

